# Localizing Non-Retinotopically Moving Objects

**DOI:** 10.1371/journal.pone.0053815

**Published:** 2013-01-14

**Authors:** Yuki Yamada, Takahiro Kawabe

**Affiliations:** 1 Research Institute for Time Studies, Yamaguchi University, Yamaguchi, Yamaguchi, Japan; 2 Faculty of Human-Environment Studies, Kyushu University, Fukuoka, Fukuoka, Japan; 3 Human Information Science Laboratory, NTT Communication Science Laboratories, Atsugi, Kanagawa, Japan; Ecole Polytechnique Federale de Lausanne, Switzerland

## Abstract

How does the brain determine the position of moving objects? It turns out to be rather complex to answer this question when we realize that the brain has to solve the motion correspondence problem in two kinds of reference frames: Retinotopic and non-retinotopic ones. We show that visual objects are mislocalized along a non-retinotopic motion direction. Observers viewed two successive movie frames each consisting of an outlined square and two target elements inside the square. In the non-retinotopic condition the elements as well as the square moved vertically while two bars also centripetally or centrifugally moved. In the retinotopic condition the vertical movement of them was removed from the stimuli. The task of the observers was to judge a relative position of the elements. Consequently, the elements were mislocalized in the direction of both retinotopic and non-retinotopic motion, although the mislocalization was significantly larger in the retinotopic than in the non-retinotopic conditions. The results suggest that non-retinotopic as well as retinotopic motion processing contributes to the determination of perceived positions of moving objects.

## Introduction

The initial and final positions of moving objects are often mislocalized. The initial position of a moving element is displaced in the direction of the motion, known as the Fröhlich effect [1], [2]. In a similar vein, a target flash is mislocalized toward a trailing task-irrelevant flash [3]. Moreover, not only the initial position but also the final position of a moving element is displaced in the direction of the motion [4]–[6].

**Figure 1:**
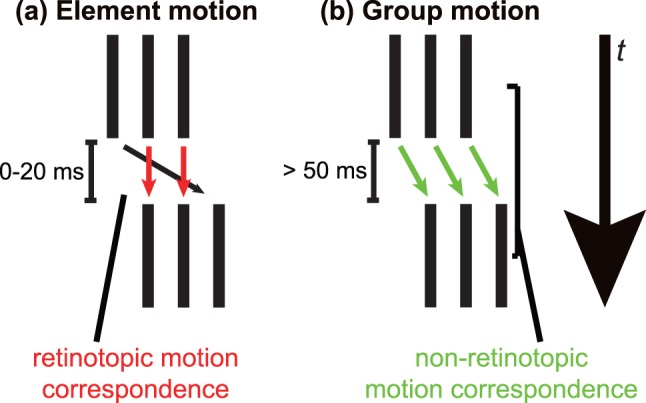
A schematic explanation for the perceived motion correspondence in the Ternus display: (a) element motion and (b) group motion. The small arrows represent the correspondences of visual elements between temporal frames.

We were interested in whether non-retinotopic motion processing also took part in the mislocalization of the initial and final positions of moving objects. Previous studies using the Ternus display have suggested that motion correspondence between visual elements can be determined retinotopically or non-retinotopically depending on the stimulus onset asynchrony (SOA) between frames [7]. When a temporal interval between two successive frames of visual elements is short (<30 ms), motion correspondence is retinotopically determined (Figure 1A), and this results in an element motion percept [8], [9]. On the other hand, when the temporal interval between frames is sufficiently long (>50 ms), motion correspondence of visual elements is non-retinotopically determined (Figure 1B) and this results in a group motion percept [8], [9]. Previous studies using the Ternus display examined when a non-retinotopic frame of reference influences some kinds of visual processing, such as motion, form, size, and attention processing [10]–[16]; Form, and attention processing occur non-retinotopically, whereas motion and tilt aftereffects occur in a retinotopic processing [11]. Moreover, non-retinotopic motion correspondence requires more attentional resources than retinotopic one [17].

The present study aimed at examining whether non-retinotopic motion correspondence caused the mislocalization of the initial (Experiment 1) and final positions (Experiment 2) of moving objects. For each experiment, we were interested in the following two issues. First, we wanted to check whether the non-retinotopic motion could induce the mislocalization of the elements. Second, we tried to look at whether the magnitude of the mislocalization varied with type of motion correspondence. If the mislocalization of the initial and final positions were determined based only on retinotopic processing such as attention shift in the direction of retinotopic motion [2], [18]–[19], the mislocalization would occur only when the motion correspondence was established retinotopically. Otherwise, the mislocalization would occur also when the motion correspondence was established non-retinotopically.

## Methods

### Ethics Statement

The experiments were conducted according to the principles laid down in the Helsinki Declaration. Written informed consent was obtained from all participants except the authors after the nature and possible consequences of the study were explained to them. The ethical committee of the Kyushu University approved the protocol.

### Experiment 1

#### Observers

Six observers including the authors participated in this experiment. The observers other than the authors were naive as to the purpose of this experiment, and all reported that they had normal or corrected-to-normal visual acuity.

#### Apparatus

Stimuli were presented on a 19-inch CRT monitor (RDF193H, Mitsubishi, Japan) with a resolution of 1024 × 768 pixels, and a 100 Hz refresh rate. The presentation of stimuli and collection of data were controlled by a computer (Mac Pro, Apple). The observer’s visual field was fixed using a chin-head rest, at a viewing distance of 60 cm and size information in visual angle described here was based on this viewing distance.

#### Stimuli

Stimuli consisted of a fixation point, a square frame, two peripheral bars, and two more central target elements. They were displayed on a gray background (31.3 cd/m^2^). The fixation point was a small white circle (58.9 cd/m^2^) with a radius of 0.05 deg and was presented in the center of the display. The square frame was a white square outline (58.9 cd/m^2^) that had a side of 2.8 deg and a border width of 0.05 deg. The target elements were green vertical rectangles (CIE:.28/.59, 59.2 cd/m^2^) with height and width of 0.24 deg × 0.05 deg and the peripheral bars were white vertical rectangles (58.9 cd/m^2^) with height and width of 0.24 deg × 0.19 deg. The peripheral bars were diagonally presented at top-left/bottom-right (i.e., left diagonal) or top-right/bottom-left (i.e., right diagonal) positions in the square frame and were vertically and horizontally displaced 3.8 deg each other. Each of the target elements was presented 1.9 deg above and below of the center of the square frame, respectively.

#### Procedure

**Figure 2:**
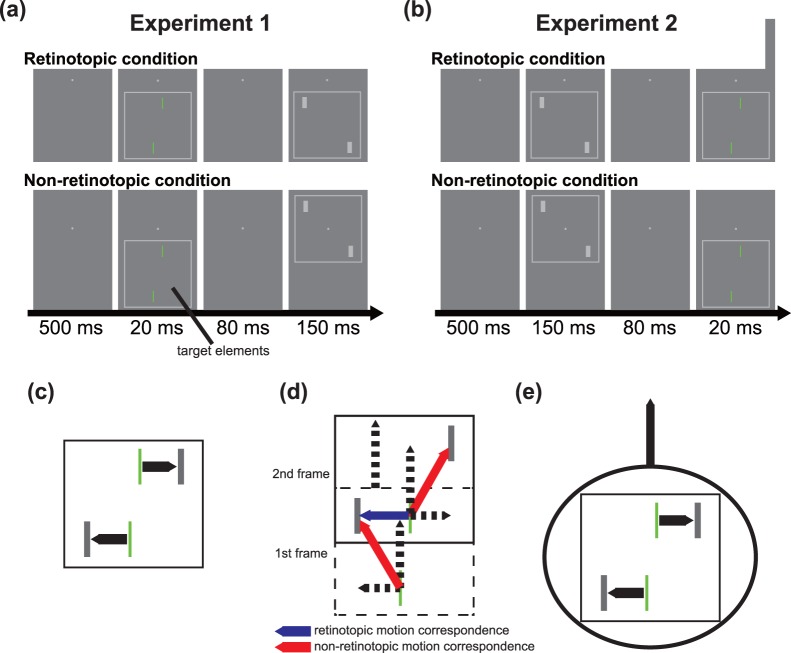
Stimuli. (a, b) Schematic representations of the time course of the stimuli used in (a) Experiment 1 and (b) Experiment 2. In the non-retinotopic condition, the visual target elements moved synchronously with the frame stimulus. (c) Motion vectors in the retinotopic condition. In this situation, there is no common component in decomposed motion vectors between bars and thus perceived motion direction is defined by retinotopic motion correspondence (the solid vectors) that is dependent on spatiotemporal proximity between the bars. (d) Decomposed motion vectors (the dashed vectors) in the non-retinotopic condition in Experiment 1. The blue solid vector represents retinotopic motion correspondence between the frames that is dependent on spatiotemporal proximity between the bars. The red solid vectors represent non-retinotopic motion correspondence between the frames. In this situation, the common (vertical) component served as a reference frame and the residual (horizontal) vectors define the perceived direction of non-retinotopic motion. (e) The resultant perceived motion direction in the non-retinotopic condition. Horizontal non-retinotopic motion (represented by the horizontal solid vectors) occurs with vertical group motion of the elements and the frame stimulus (represented by the vertical solid vector and the circle). The length of the horizontal motion vectors in motion correspondence in the retinotopic condition (shown in c) is equal to the horizontal components in motion correspondence in the non-retinotopic condition (shown in d). That is, the intensity of horizontal non-retinotopic motion is equal to horizontal retinotopic motion.

Figure 2A shows a time course of the stimulus presentation in Experiment 1. The experiment was conducted in a darkened room. The observer initiated each trial by pressing the spacebar on a computer keyboard. The fixation point was presented throughout the experiment. After a delay of 500 msec, the square frame and the target elements were presented for 20 msec. The square frame was placed 1.9 deg below the center of the display. Then, a blank screen was inserted for 80 msec. Subsequently, the square frame and the peripheral bars were presented for 150 msec. In the retinotopic condition, the position of the second square frame was the same as the first square frame (Movie S1). In the non-retinotopic condition, the second square frame was placed 1.9 deg above the position of the first square frame (i.e., at the center of the display) (Movie S2). The horizontal offset of the target elements was varied with the randomly interleaved double staircase method (one-up/one-down). Step size of each staircase was varied with the number of reversal: 0.19 (until the first reversal), 0.10 (until the second reversal), and 0.05 deg (after the third reversal). One staircase started at the locations closest to the peripheral bars (i.e., the offset of +0.37 deg), whereas the other interleaved staircase started at the locations farthest from the peripheral bars (i.e., the offset of −0.37 deg). Each staircase ended after 20 reversals of the staircase. No explicit feedback for the correctness of responses was provided. Observers were asked to judge a horizontal misalignment of the green target elements by indicating whether the upper element was perceived being left or right of the lower element, after the stimulus presentation was finished. There were 2 variables: position of peripheral bars (left and right diagonals) and retinal position (retinotopic and non-retinotopic), and each variable was blocked. The order of the blocks was randomized across observers.

### Experiment 2

#### Observers

Eight observers including one of the authors (YY) participated in this experiment. The observers other than YY were naive as to the purpose of this experiment, and all reported that they had normal or corrected-to-normal visual acuity.

#### Apparatus, stimuli, and procedure

Experiment 2 was identical to Experiment 1 except for the followings (Figure 2B): After a delay of 500 msec from pressing the spacebar, the first square frame and the peripheral bars were presented for 150 msec. Then, a blank screen was inserted for 80 msec. Subsequently, the second square frame and the target elements were presented for 20 msec. In the retinotopic condition, the positions of both the first and second square frames were 1.9 deg below the center of the display (Movie S3). In the non-retinotopic condition, the first square frame was placed at the center of the display and the second square frame was placed 1.9 deg below the position of the first square frame (Movie S4). Observers were asked to judge a horizontal misalignment of the green target elements in the second square frame by indicating whether the upper element was perceived being left or right of the lower element, after the stimulus presentation was finished.

## Results

### Experiment 1

**Figure 3:**
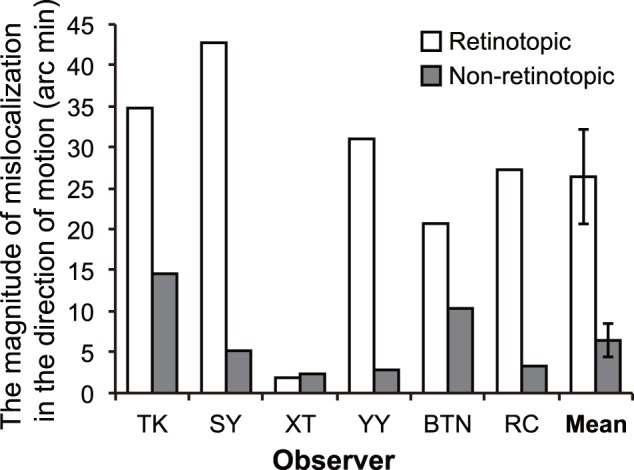
The results of the Experiment 1. Individual and mean data are shown. Error bars denote standard errors of the mean.

The average for the last 6 reversals in each condition was used as the estimated PSE. We calculated the difference between the estimated PSEs of left and right diagonal conditions as the mislocalization magnitude for the initial position of the target elements in the direction of apparent motion. For example, the PSE for observer RC showed that the upper target −12.69′ offset rightward (i.e., 12.69′ offset leftward) from the lower target in the left diagonal condition and the upper target 14.57′ offset from the lower target in the right diagonal condition of the retinotopic condition. At this stage, positive values represent rightward mislocalization. Then, the difference of these two conditions, 27.26′, was finally used for statistical analysis. Here, positive values represent the mislocalization in the direction of apparent motion. The magnitude of mislocalization for each observer and group mean of it are shown in Figure 3. All observers showed the positive magnitude of mislocalization both in the retinotopic and non-retinotopic conditions. One-sample *t*-tests revealed that the initial position shift in the direction of apparent motion was significantly larger than 0 both in the retinotopic [*t*(5) = 4.59, *p*<.006, Cohen’s *d* = 2.65] and non-retinotopic conditions [*t*(5) = 3.18, *p*<.03, Cohen’s *d* = 1.83]. A two-tailed paired *t*-test revealed that the mislocalization magnitude in the retinotopic condition was significantly larger than that in the non-retinotopic condition [*t*(5) = 3.64, *p*<.02, Cohen’s *d* = 1.89]. These results showed that the initial position of apparent motion was significantly displaced in the direction of motion even when the motion correspondence was determined in a non-retinotopic fashion. The mislocalization of the target elements in retinotopic motion was significantly larger than the one in non-retinotopic motion. These significant differences had large effect sizes.

### Experiment 2

**Figure 4:**
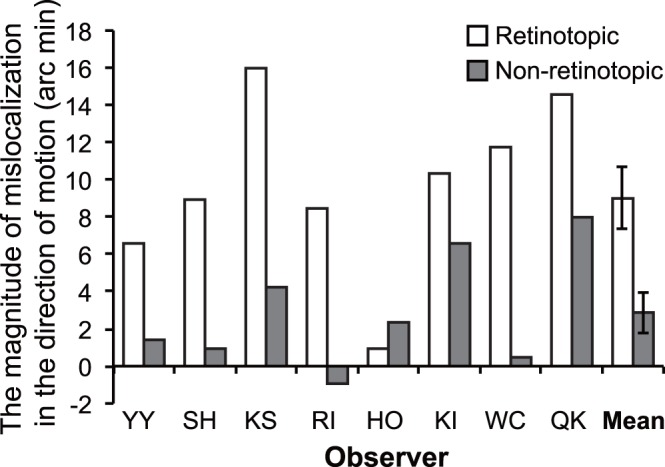
The results of the Experiment 2. Individual and mean data are shown. Error bars denote standard errors of the mean.

The results of Experiment 2 are shown in Figure 4. As in Experiment 1, we estimated the PSE and calculated the difference between the estimated PSEs of left and right diagonal conditions as the mislocalization magnitude of the target elements in the direction of apparent motion. All the observers other than one observer showed the positive mislocalization magnitudes both in the retinotopic and non-retinotopic conditions. One-sample *t*-tests revealed that the mislocalization magnitude was significantly larger than 0 both in the retinotopic [*t*(7) = 5.80, *p*<.0007, Cohen’s *d* = 2.69] and non-retinotopic conditions [*t*(7) = 2.61, *p*<.04, Cohen’s *d* = 1.31]. A two-tailed paired *t*-test revealed that the mislocalization magnitude in the retinotopic condition was significantly larger than that in the non-retinotopic condition [*t*(7) = 4.44, *p*<.004, Cohen’s *d* = 1.53]. These results showed that the final position of apparent motion was also significantly displaced in the direction of motion even when the motion correspondence was determined in a non-retinotopic fashion. As in Experiment 1, the final position shift in retinotopic motion was significantly larger than that in non-retinotopic motion. These significant differences had large effect sizes.

## Discussion

The goal of the present study was to investigate whether the initial and final position of moving objects was mislocalized in the direction of non-retinotopic as well as retinotopic motion. The results of Experiments 1 and 2 showed that the non-retinotopic motion caused the significant mislocalization of the target elements. These results suggest that non-retinotopic motion processing also takes part in the determination of the location of visual objects.

What underlies the mislocalization of visual objects due to non-retinotopic motion? For the mislocalization of the objects due to retinotopic motion, attention shift has been proposed as the main cause of the mislocalization [2], [18]–[19]. Fröhlich effect, which was a representative phenomenon for the mislocalization of the initial position of a moving object, has been explained by the combination of attention shift and metacontrast [2]. That is, the representation of the initial position of a motion stimulus is suppressed by metacontrast from the subsequent stimulus, and attention does not intensify the representation at the initial position due to its shift in the motion direction, biasing the judgment of the initial position in the motion direction. The forward displacement of a final position of a moving stimulus is also explained by attention shift in the motion direction [18]–[19]. Attention shifts in the direction of a moving stimulus and overshoots beyond the actual final position of the stimulus. Mental extrapolation for the stimulus position occurs in the direction of attention shift, resulting in the forward displacement. Moreover, a recent study demonstrated that the attention shift was possible within a moving non-retinotopic frame of reference [12]. Thus, the attention shift within a non-retinotopic frame of reference might cause the forward and backward mislocalization of the target elements in apparent motion.

Non-retinotopic representation of motion has been depicted based on the idea of motion vector decomposition [20]–[23]. In general, a retinotopic motion vector can be decomposed into two orthogonal vector components (i.e. horizontal and vertical components). Figure 2D shows how the motion signals in our non-retinotopic stimuli can also be decomposed into the orthogonal vector components. As shown in Figure 2D, vertical vector components are common in the elements, serving as a reference frame for determining motion correspondence; the vertical vector component as a reference frame can inhibit the retinotopic motion correspondence between the upper bar in the first frame and the lower bar in the second frame. The residual horizontal vector component in each element defines non-retinotopic motion of the bar within the reference frame. These horizontal components of motion vectors would contribute to the target mislocalization in the non-retinotopic condition. However, in the present experiments the mislocalization magnitude in the non-retinotopic condition was significantly smaller than that in the retinotopic condition. This might be because of the competition between retinotopic and non-retinotopic motion correspondences in our stimulus configuration, wherein besides the non-retinotopic correspondence, the retinotopic motion correspondence could be established between the upper bar in the first display and the lower bar in the second display due to spatiotemporal proximity between the bars. Here we assume that the non-retinotopic motion correspondence was relatively dominant over the retinotopic one. This assumption is based on the following two ideas; first, as described above, the vertical motion vector common in the elements could suppress the retinotopic correspondence, and simultaneously enhance the non-retinotopic correspondence. Second, because the vertical motion vector was common to the motion vector of frame stimulus, the motion vector could presumably serve as a reference frame for the motion mechanism to choose the non-retinotopic motion correspondence as a likely one. The direction of attention shift is assumingly dependent on perceived motion direction as a product of solving the competitive motion correspondence. Because the non-retinotopic motion correspondence was predominant over the retinotopic one, attention shift also possibly occurred in the direction of the non-retinotopic motion correspondence more often than in the direction opposite to the non-retinotopic motion correspondence (i.e., in the direction of the retinotopic motion correspondence). Consequently, a weaker but significant mislocalization of bars might occur in the non-retinotopic motion direction. Further experiments that confirm the perceived direction of apparent motion in our stimuli will be needed to examine this explanation.

Otherwise, the smaller magnitude of mislocalization in the non-retinotopic condition may be explained with the suppression of apparent motion within the frame due to the motion of the frame. The visual awareness for feature changes inside a moving object is suppressed [24]. Hence, visual awareness for position changes (i.e., apparent motion) inside the square frame might be suppressed during the frame’s motion, and this possibly resulted in the small magnitude of mislocalization.

Attentional repulsion may account for the results of the forward mislocalization of the target elements observed in Experiment 2. Previous studies using a spatial cueing paradigm have suggested that a transient flash is mislocalized away from a pre-cued position [25]–[27]. In Experiment 2, it was possible that peripheral bars at the first frame captured attention at their positions, and repelled the target elements. Cueing can occur within a non-retinotopic frame of reference [12], and therefore the attentional repulsion account is also applicable to the results in the non-retinotopic condition.

Consistent with the previous findings, the present study suggests that higher-order motion affects the perceived position. For example, second-order motion [28] and transformational apparent motion [29] can induce position shifts of stationary visual elements. In addition to these previous studies, the present study suggests that the perceived position is determined after the non-retinotopic motion processing is completed. However, it is still unclear whether the non-retinotopic motion can bias the perceived position of nearby stationary visual elements as the retinotopic motion can do so [30], [31]. Future studies may clarify the extent to which non-retinotopic motion processing contributes to localization processing. This is necessary for a complete comprehension of visual processing underlying the location perception.

## Supporting Information

Movie S1 **The initial position shift in the retinotopic motion direction.**
(GIF)

Movie S2 **The initial position shift in the non-retinotopic motion direction.**
(GIF)

Movie S3 **The final position shift in the retinotopic motion direction.**
(GIF)

Movie S4 **The final position shift in the non-retinotopic motion direction.**
(GIF)
