# Supplementary figures and images for: Localizing Non-Retinotopically Moving Objects
Source: PLoS One. 2013 Jan 14;8(1):e53815. doi: 10.1371/journal.pone.0053815 (PMC3544746; doi:10.1371/journal.pone.0053815)

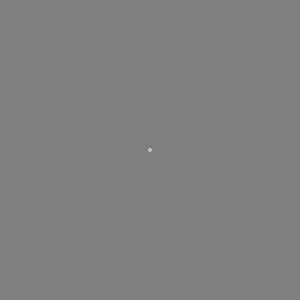

Supplement: Movie S1 — The initial position shift in the retinotopic motion direction. (GIF) [file pone.0053815.s001.gif]

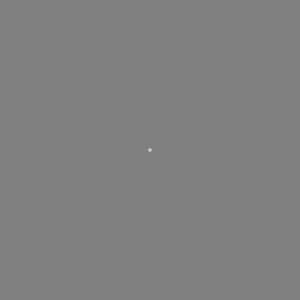

Supplement: Movie S2 — The initial position shift in the non-retinotopic motion direction. (GIF) [file pone.0053815.s002.gif]

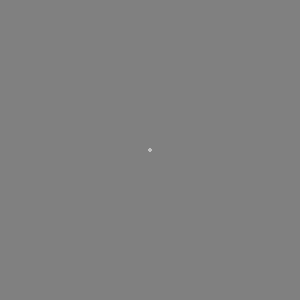

Supplement: Movie S3 — The final position shift in the retinotopic motion direction. (GIF) [file pone.0053815.s003.gif]

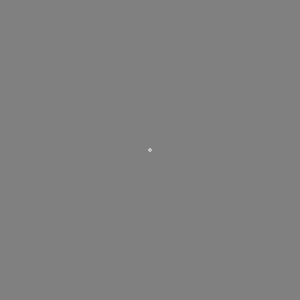

Supplement: Movie S4 — The final position shift in the non-retinotopic motion direction. (GIF) [file pone.0053815.s004.gif]
